# Isolation, identification, and characterization of resistant bacteria to antibiotics from pharmaceutical effluent and study of their antibiotic resistance

**DOI:** 10.3389/fmicb.2023.1307291

**Published:** 2023-12-27

**Authors:** Md. Shahin Mahmud, Md. Aoulad Hosen, Md. Ishaq Hossion, Md. Shiblee Sadik Sabuj, Nazmi Ara Rumi, Md. Khaled Hossain, Musaab Dauelbait, Hiba-Allah Nafidi, Turki M. Dawoud, Muhammad Ibrahim, Mohammed Bourhia

**Affiliations:** ^1^Department of Microbiology, Faculty of Veterinary and Animal Science, Hajee Mohammad Danesh Science and Technology University, Dinajpur, Bangladesh; ^2^Department of Scientific Translation, Faculty of Translation, University of Bahri, Khartoum, Sudan; ^3^Department of Food Science, Faculty of Agricultural and Food Sciences, Laval University, Quebec City, QC, Canada; ^4^Department of Botany and Microbiology, College of Science, King Saud University, Riyadh, Saudi Arabia; ^5^Department of Biosciences, COMSATS University Islamabad, Sahiwal Campus, Sahiwal, Pakistan; ^6^Department of Chemistry and Biochemistry, Faculty of Medicine and Pharmacy, Ibn Zohr University, Laayoune, Morocco

**Keywords:** *Pseudomonas* spp., drug-resistant, isolation, antibiotics, bacteria

## Abstract

Pharmaceutical effluents primarily enter aquatic environments through the discharge of treated and untreated wastewater from various sources, including hospitals, pharmaceutical manufacturing facilities, and households. Microbes sourced from pharmaceutical effluents such as *Pseudomonas* spp. pose a significant public health concern because of their high levels of resistance to multiple drugs and extreme multidrug resistance. Therefore, the present study was conducted for the isolation, identification, and molecular characterization of selected isolates from pharmaceutical effluents and also determined their antibiotic sensitivity patterns. From June 2016 to March 2017, a study was conducted on four well-known pharmaceutical companies specializing in antibiotic production in Dhaka and Gazipur. Four wastewater samples were collected from various origins and then brought to the Bacteriology laboratory for microbiological examination. Twelve pure isolates were obtained and characterized through cultural and biochemical tests while molecular identification of *Pseudomonas* spp. was performed using the 16S rRNA gene sequence. Twelve commercially available antibiotics were used for antibiotic sensitivity tests using Kirby-Bauer disk diffusion methods. We isolated the most predominant isolates, *Pseudomonas aeruginosa* (41.67%), followed by *Bacillus* spp. (33.33%) and *Staphylococcus* spp. (25%) respectively. Among 12 antibiotics, ciprofloxacin is 100% sensitive against *P. aeruginosa,* while the remaining 11 antibiotics are 100% resistant. *Bacillus* spp. showed 100% resistance to all antibiotics while 50% sensitive to vancomycin and 100% to chloramphenicol, respectively. *Staphylococcus* spp. was 100% resistant to all antibiotics. Our research suggested that *P. aeruginosa* is the reservoir of antibiotic resistance genes and spreads disease to humans from the environment. The findings of this study, i.e., the isolation, identification, and characterization of antibiotic-resistant bacteria from pharmaceutical effluent have highlighted, comprehended, and mitigated the dissemination of antibiotic resistance and opportunistic bacteria.

## Introduction

Pharmaceutical discharges are the waste products of the pharmaceutical company generates during a drug’s manufacturing process (Geta and Kibret, 2022). The pharmaceutical industry’s effluents contain antibiotics, prescription, and non-prescription drugs (Balasa et al., 2021). Antibiotic resistance and hospital-acquired diseases can result from chronic antibiotic exposure. Antimicrobial disposal into aquatic habitats has become an increasingly prevalent serious environmental issue because of its potential harm to the ecosystem and human health (Terfa Kitila et al., 2018). In poor waste management systems, most antibiotics used for treating humans, livestock animals, and plants are excreted into the environment as their unchanged precursors via wastewater effluent discharge, runoff from agricultural or human waste-applied land, and leaching (Terfa Kitila et al., 2018). During the last 15 years, pharmaceuticals have been receiving great attention, as they are highly biologically active compounds. Wastewater Treatment Plants (WWTP) release veterinary and human antibiotic residues into the environment, which may negatively impact water quality, ecosystems, and human and animal health (Rutgersson et al., 2014; Angelidaki et al., 2020). Antibiotic residues in wastewater effluents pose a serious environmental concern as they are recalcitrant against various wastewater treatment processes. If antibiotics are dumped into the water, it could hurt bacterial groups and cause them to become resistant to numerous antibiotics. Antibiotic resistance genes (ARGs) are found in many polluted aquatic habitats and are responsible for the further spread of antibiotic resistance traits. Suzuki et al. (2017) reported that antimicrobial resistance in water ecosystems had been associated with the disposal of multiple antibiotics. Antibiotic-resistant microorganisms in pharmaceutical wastewater can spread drug resistance to pathogens (Dozie-Nwachukwu, 2016). The presence of resistant microorganisms in pharmaceutical effluents is a global public health issue, and antibiotic resistance is a public health significance. Nearly 2 million Americans and 25,000 Europeans are afflicted with antibiotic-resistant bacteria yearly, and 23,000 die directly from these infections (Brinkac et al., 2017). Lin et al. (2017) predicted that if we do not take effective action, pathogenic bacteria will kill 700,000 people each year, and the number could be 10 million by 2050 globally. Thus, antibiotic resistance also appears in pathogenic bacteria and aquatic and terrestrial organisms. Bacteria can be intrinsically resistant to one or more antibiotics. Antibiotic resistance can be reduced but not stopped since bacteria evolve. New medications and diagnostic techniques to track antibiotic resistance will always be needed (US Department of Health and Human Services, 2017). Therefore, this research aimed to identify resistant bacteria from pharmaceutical effluent and determine their antibiotic resistance patterns.

## Materials and methods

### Study area and study time period

The current investigation focused on the wastewater discharged by antibiotic-producing pharmaceutical factories in Dhaka and Gazipur districts, Bangladesh. From June 2016 through March 2017, the laboratory work was carried out in the Department of Microbiology, Faculty of Veterinary and Animal Science, Hajee Mohammad Danesh Science & Technology University (HSTU), Dinajpur.

### Sample collection and processing

A total of four pharmaceutical effluent samples were collected from different units of antibiotic-producing areas and transferred to the Bacteriology laboratory for Microbiology analysis. Using the standard method, wastewater samples were collected into sterile plastic containers and processed within 2–3 h from the collection time (Rabiu and Falodun, 2017).

### Isolation and identification of isolates

The pharmaceutical wastewater was mixed with distilled water and prepared with 10^−1^ to 10^−7^ dilution. After dilution, 0.1 mL of diluents was spread from each dilution tube to different culture plates, such as nutrient agar, Mac Conkey agar, and EMB agar. A sterile glass spreader spread the samples on culture plates aseptically. Finally, all Petri dishes were incubated at 37°C overnight. After overnight incubation, distinct bacterial colonies were picked and again subcultured for pure isolation on selective media (Rabiu and Falodun, 2017). A series of biochemical tests, such as oxidase, catalase, MR-VP, TSI, etc., were used to identify the isolated bacteria (Rabiu and Falodun, 2017). All bacteriological media were purchased from Hi Media Laboratories Limited, India.

### Molecular identification of the isolates

Biochemical tests identified *Pseudomonas* spp., *Staphylococcus* spp., and *Bacillus* spp. from wastewater of antibiotic-producing pharmaceuticals. For sequencing, DNA was extracted with an Eppendorf tube using the boiling and freezing method (Dri-block DB.2A, Techne, SA) at 100°C for 10 min. Then the samples were centrifuged at 11,000 rpm at 25°C for 3 min and transferred to an icebox. After centrifugation, the top supernatant was transferred into new tubes and processed for DNA template for PCR assay (Hollman et al., 2021). 16S rRNA gene was amplified with F27 (5′AGAGTTTGATCCTGGCTCAG3′) and R1492 (5′TACCTTGTTACGACTT3′) primers with 50 μL PCR reaction volume (Luczkiewicz et al., 2015). PCR conditions were: 95°C for 5 min, 10 cycles of 94°C for 15 s, 53°C for 30 s, and 72°C for 45 s, with a final extension cycle of 72°C for 10 min. The final PCR product was viewed in agarose gel electrophoresis and visualized and photographed by a UV transilluminator. Sequencing of the 16S rRNA was done by chain termination method (Sanger and Coulson method) with Genetic Analyzer 3130 (Applied Biosystems) in the organization of the National Institute of Biotechnology, Savar, Dhaka. The MEGA6 program measured a phylogenetic tree by applying the neighbor-joining method of 1,000 replicates used for bootstrapping (Tamura et al., 2013).

### Antibiotic susceptibility tests

According to CLSI, agar disk diffusion techniques were used to determine the antibiotic sensitivity patterns of isolates on Muller-Hinton agar plates [Clinical and Laboratory Standards Institute (CLSI), 2014]. A total of 12 commercially available antibiotics, including Amoxycillin (10 μg), Cefaclor (30 μg), Cefixime (5 μg), Cefotaxime (30 μg), Chloramphenicol (30 μg), Vancomycin (30 μg), Tobramycin (10 μg), and Penicillin (10 μg) were used. All antibiotic disks were purchased from Oxoid Limited, United Kingdom. After biochemical identification, pure colonies of isolates were spread on Muller-Hinton agar and selected antibiotic disks were placed using sterile forceps. Finally, the plates were incubated at 37°C for 24 h and the diameter zone of inhibition was measured on a millimeter scale. Moreover, according to Mir et al. (2022), the MAR index was calculated and interpreted by using the formula: *a*/*b*, Where, *a* indicates the number of antibiotics to which an isolates was resistant, and *b* indicates the total number of tested antibiotics.

## Results and discussion

**Figure 1:**
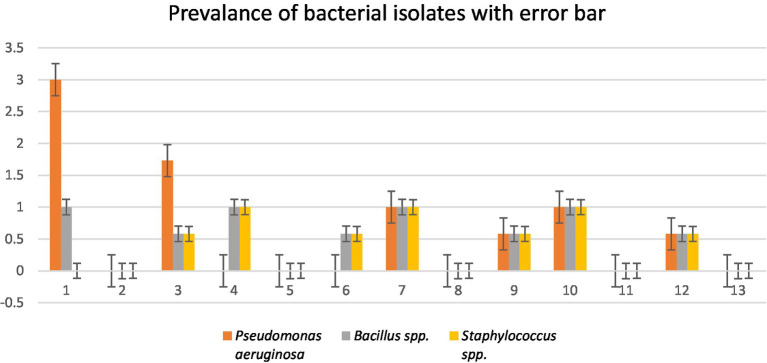
Prevalence of isolates from wastewater samples. Sixteen distinct colony types were processed for culturing and re-culturing in order to get the pure culture and 12 pure cultures were chosen based on staining and morphological characteristics observed on various selected media to aid in identification (Figure 2).

A total of four wastewater samples were collected from antibiotic-producing Effluent Treatment Plants (ETP). Total viable counts in dilution 10^7^ from sample 1 were 4.3 × 10^9^ cfu/mL; followed by 4.6 × 109 cfu/mL from sample 2, 4.9 × 10^9^ cfu/mL from sample 3, and 6.7 × 10^9^ cfu/mL from sample 4 were calculated;. A total of 12 pure isolates including *Pseudomonas aeruginosa* (41.67%), *Bacillus* spp. (33.33%), and *Staphylococcus* spp. (25%) were identified from the four different sources (Table 1; Figure 1).

**Table 1:** Prevalence of isolates from wastewater samples from different sources.

Bacterial isolates	Wastewater (Sample 1)	Wastewater (Sample 2)	Wastewater (Sample 3)	Wastewater (Sample 4)	Percentage (%)
*Pseudomonas aeruginosa*	3	0	1	1	5 (41.67%)
*Bacillus* spp.	1	1	1	1	4 (33.33%)
*Staphylococcus* spp.	0	1	1	1	3 (25%)
Total isolates	4	2	3	3	12 (100%)

**Figure 2:**
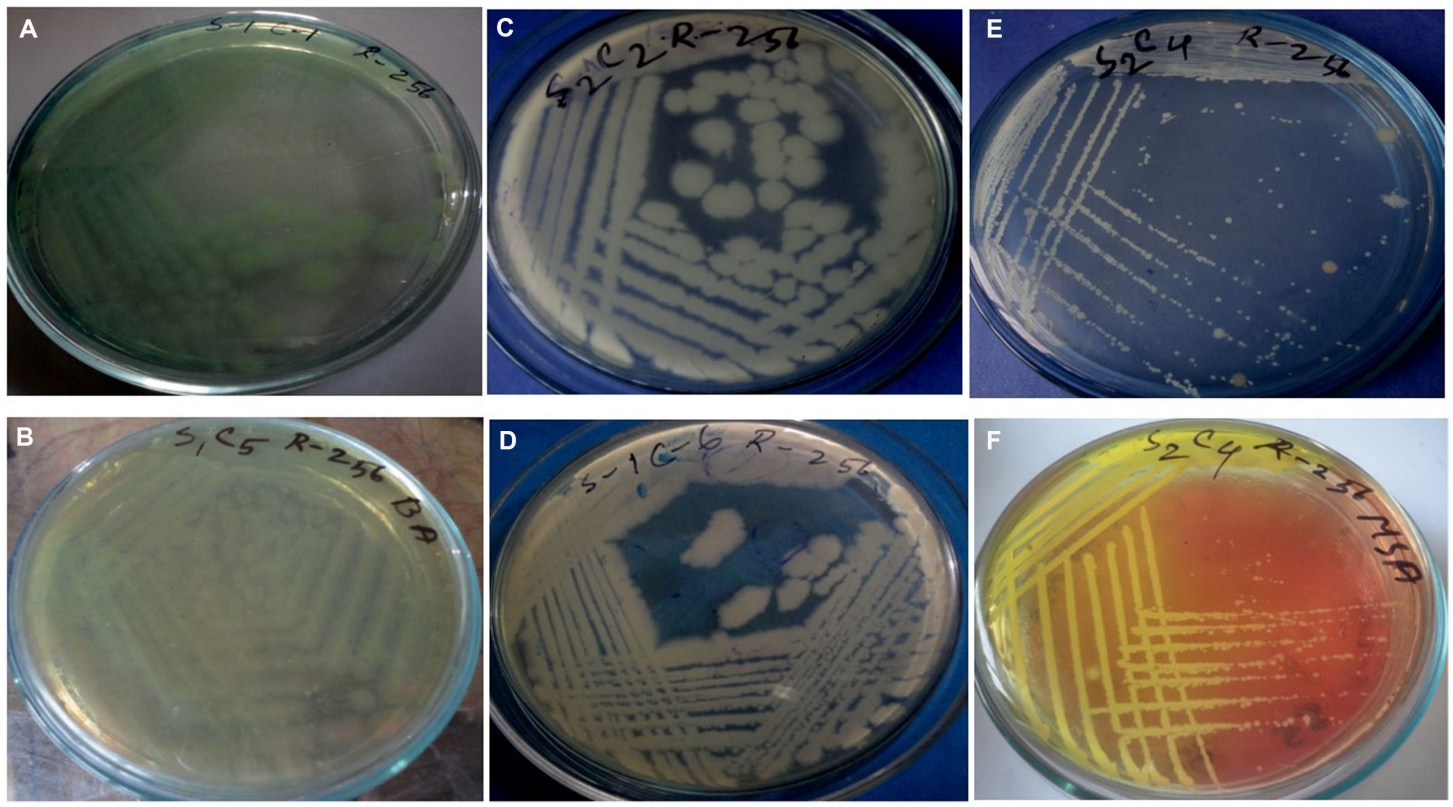
Cultural characteristics of *Pseudomonas aeruginosa* on **(A)** Nutrient agar, **(B)** Cetrimide agar; *Bacillus* spp. on **(C,D)** Nutrient agar; *Staphylococcus* spp. on **(E)** Nutrient agar, and **(F)** MSA.

A group of biochemical tests such as catalase, MR-VP, indole, citrate utilization, MIU, and TSI confirmed the bacterial identification (Table 2). *Pseudomonas aeruginosa* was the predominant isolate in our study, accounting for 60% in sample number 1. In this study, most of the isolates were related to *P. aeruginosa*, and representative isolates were identified by molecular method. To determine the genetic diversity and evolutionary relationship between *P. aeruginosa* strain S1C1 and those in other publicly available databases “*n* = 1,059,” we conducted phylogenetic analyses using 16S rRNA with the 20 most close strains based on BLAST search similarity (Figure 2).

**Table 2:** Results of biochemical tests.

S.No.	Sample	MR	VP	Indole	Citrate utilization	MIU	TSI	Catalase
1	S_1_C_1_	−	−	−	+	−	Slant and Butt are both alkaline	+
2	S_1_C_2_	−	+	−	−	+	Slant alkaline Butt acidic	+
3	S_1_C_3_	−	+	−	+	+	Slant alkaline Butt acidic	+
4	S_1_C_5_	−	−	−	+	−	Slant and Butt are both alkaline	+
5	S_1_C_6_	−	+	−	−	+	Slant alkaline Butt acidic	+
6	S_2_C_1_	−	+	−	+	−	Slant and Butt Both alkaline	+
7	S_2_C_3_	−	+	+	+	−	Slant alkaline Butt acidic	+
8	S_2_C_4_	+	−	−	−	−	Slant and Butt are both acidic	+
9	S_3_C_1_	−	−	−	+	−	Slant and Butt are both alkaline	+
10	S_3_C_2_	−	+	−	+	+	Slant alkaline Butt acidic	+
11	S_4_C_1_	−	+	−	+	+	Slant alkaline Butt acidic	+
12	S_4_C_2_	−	−	−	+	−	Slant and Butt are both alkaline	+

MR, Methyl red; VP, Voges-Proskauer; MIU, Motility indole urease; TSI, Triple sugar iron; S, Sample; and C, Colony.

**Figure 3:**
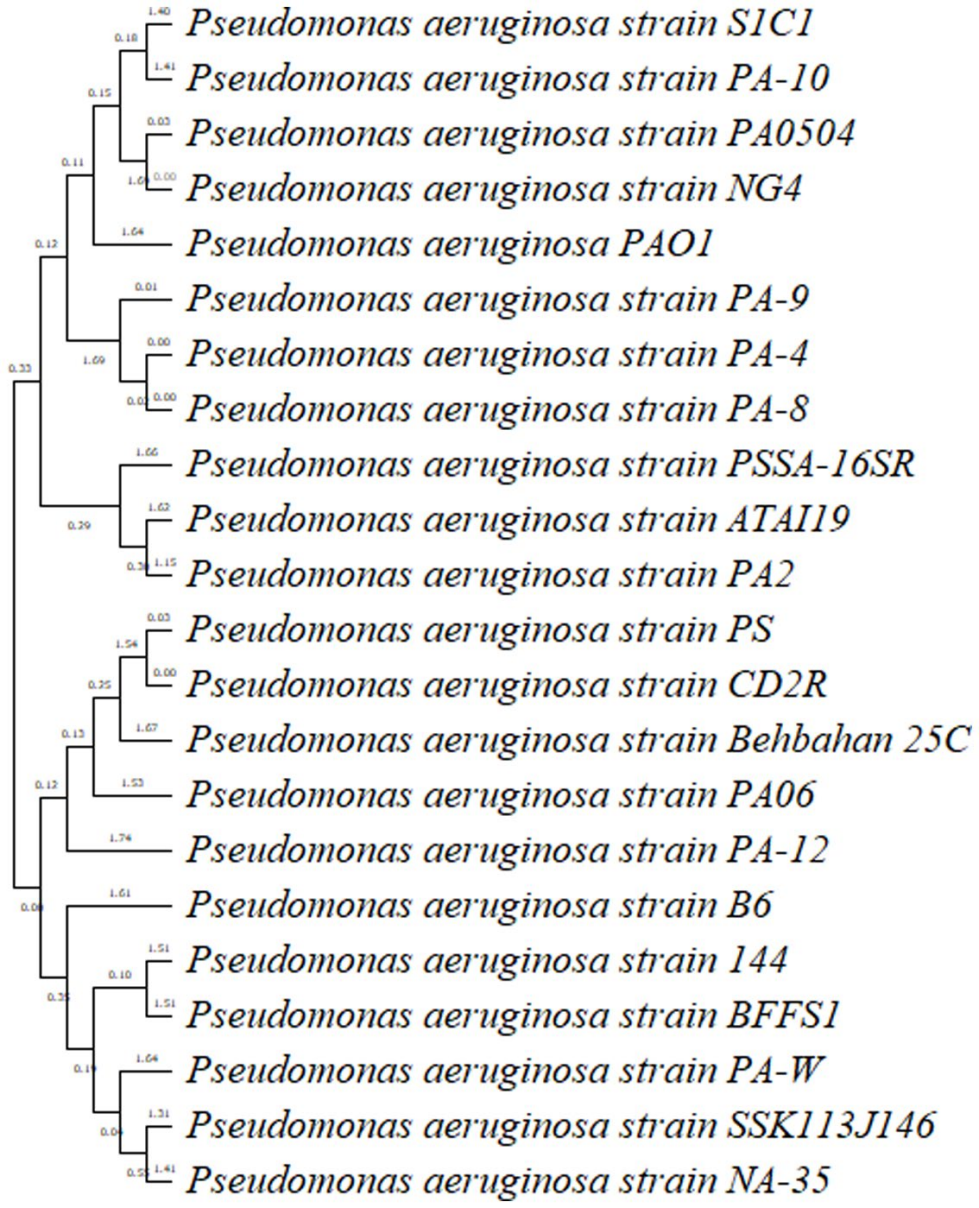
Phylogenetic tree analysis of *Pseudomonas aeruginosa strain S1C1 by* using neighbor-joining with 16S rRNA gene. Outgroup not included.

The tree derived from 16S rRNA has traditionally been used to define prokaryotic taxonomy. Overall, in BLAST analysis *P. oleovorans,* and *P. alcaligenes* were also among the top 10 species having similarity of more than 90% but *P. aeruginosa* strain S1C1 made separate branch in an evolutionary tree which was linked with a cluster having *P. aeruginosa* strain Dg-N13 and *P. aeruginosa* strain PA14. The 16S rRNA tree revealed that *P. aeruginosa* strain S1C1 seemed genetically more identical to *P. aeruginosa* strain Dg-N13 and *P. aeruginosa* strain PA14 (Figure 3). The 16 s gene sequence of strain S1C1 was submitted to the NCBI gene bank under accession No. OR029393.1, and sequencing results are present as Supplementary Table S1.

*Pseudomonas aeruginosa* strain PA10, a clinical isolate (Shaikh et al., 2022), and *P. aeruginosa* reference strain PA0504 and strain PA01 are the most extensively studied strains of *P. aeruginosa* and have been widely used as a model organism in research. Moreover, PA14 is highly virulent and can cause a range of infections, including respiratory tract infections, urinary tract infections, wound infections, and bloodstream infections. It possesses several virulent factors, such as adhesins, toxins, and secretion systems, which contribute to its pathogenicity (Mikkelsen et al., 2011). Identification of *P. aeruginosa* strain S1C1 and in the cluster similar to PA01 shows S1C1 seems to be a highly virulent strain.

**Figure 4:**
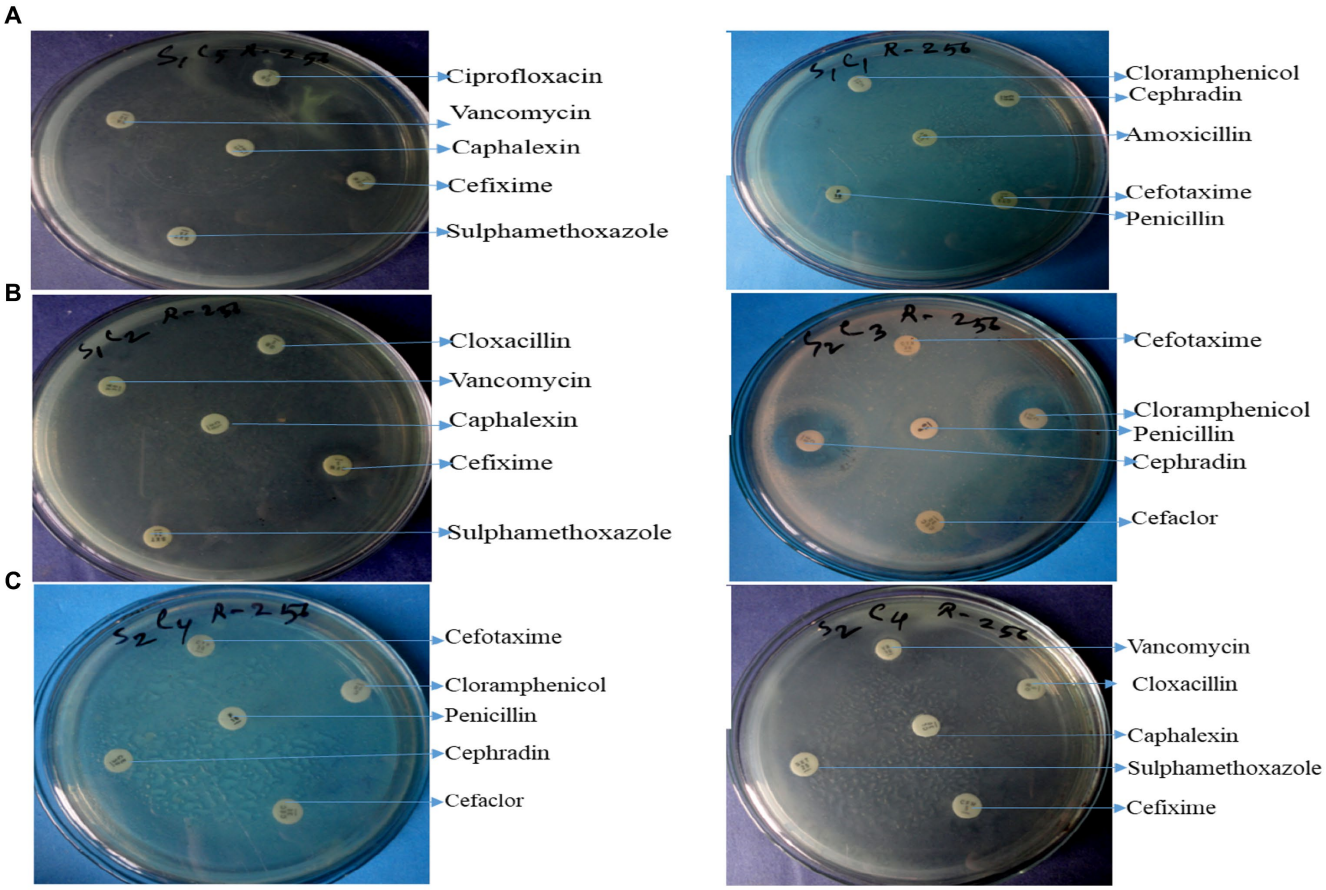
Antibiotic resistant profile of *Pseudomonas aeruginosa* **(A)**, *Bacillus* spp. **(B)**, and *Staphylococcus* spp. **(C)**.

Twelve commercially available antibiotics such as Cephalexin, Vancomycin, Cefixime, Sulphamethoxazole, Cloxacillin, Chloramphenicol, Cefotaxime, Penicillin, Cephradin, Ciprofloxacin, Amoxicillin, and Tobramycin were used to antibiotic sensitivity tests against isolated bacteria. The antimicrobial sensitivity test was performed according to the procedure Kirby-Bauer disk diffusion susceptibility test protocol suggested by Hudzicki (2009). No major variation was noticed in the sensitivity of isolates against 12 different antibiotics used. The antibiotic-resistant result is shown in Supplementary Table S3 and Figures 4A–C.

**Figure 5:**
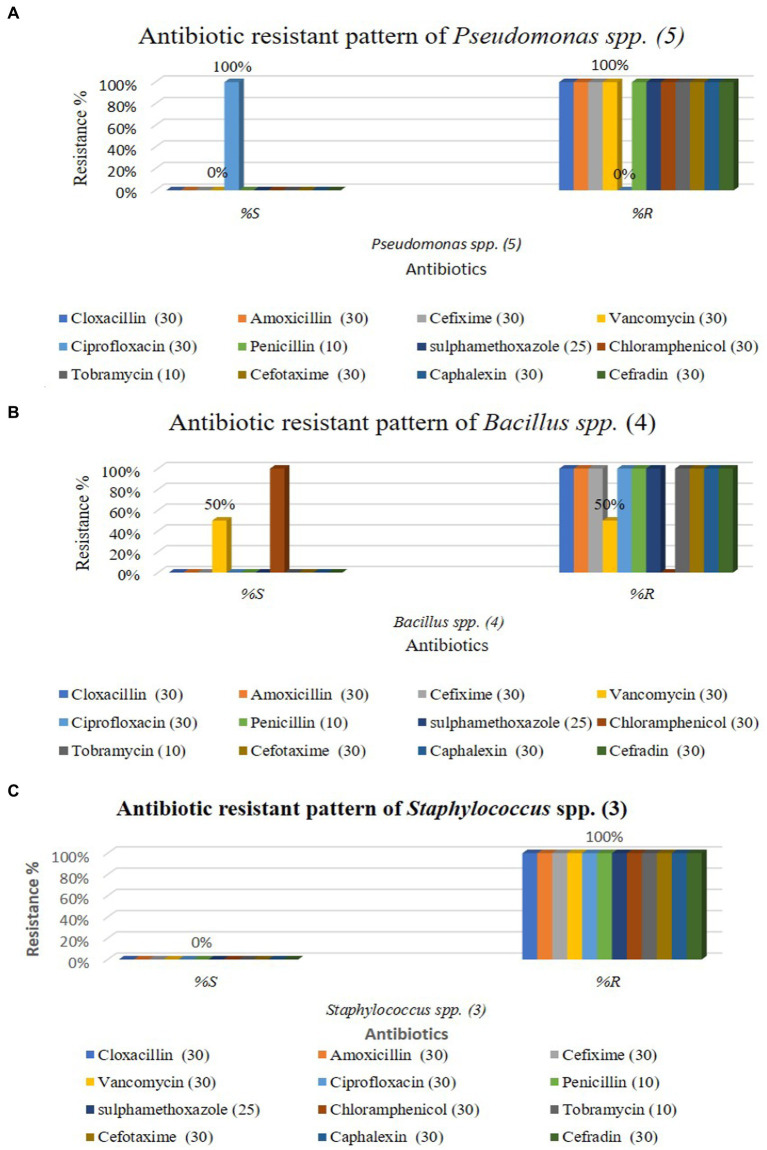
Antibiotic resistant pattern of *Pseudomonas aeruginosa* **(A)**; *Staphylococcus* spp. **(B)**; and *Bacillus* spp. **(C)**.

The graphical antibiotic-resistant results are shown in Figures 5A–C. Most of the identified isolates showed multi-drug resistance. Our study showed that *P. aeruginosa* was 100% resistant against cefotaxime, but the other author showed their study 100% sensitive to the same antibiotic (Hollman et al., 2021). Our present research revealed that *P. aeruginosa* was 100% resistant, and Bolaji et al. (2011) reported the same result in their research. Out of 12 commercial antibiotics except for ciprofloxacin, all were 100% resistant to *P. aeruginosa* (Figures 4, 5A), and the result is more or less similar to Rabiu and Falodun (2017). *Bacillus* spp. showed 50% sensitivity against vancomycin and 100% against chloramphenicol, while remaining all antibiotics showed 100% resistance (Figures 4, 5B). The intensity of the inhibition zone was measured as are described in Supplementary Table S2. The MAR Index ranged begin from 0.92 to 1.00 and the average MAR index being 0.98 in five isolates of *P. aeruginosa* (Supplementary Table S2). In Supplementary Table S3, the MAR Index ranged begin from 0.833 to 1.00 and the average MAR index being 0.93 in four isolates of *Bacillus* Spp. In Supplementary Table S4, the MAR Index ranged begin from 1.00 to 1.00 and the average MAR index being 1.00 in three isolates of *Staphylococcus* spp. (Figure 5C).

Our findings agree with the findings of Aggarwal et al. (2015) in which they isolated *Pseudomonas* spp. a strain that showed resistance to five out of eight antibiotics tested. Our result showed that all antibiotics were 100% resistant against *Staphylococcus* spp., represented in Figures 4, 5C. Similarly, Farooq et al. (Geta and Kibret, 2022) identified *Bacillus* spp., *Klebsiella* spp., *Coccobacilli*, *Staphylococcus* spp., *and Enterobacter* spp. Unidentified *Bacilli*, *Diplococcus* spp., *Micrococcus* spp., *Streptobacillus* spp., and *Shigella* spp. The *Staphylococcus* spp. showed maximum resistance (SF21) against 10 antibiotics. Tahrani et al. (Terfa Kitila et al., 2018) identified *Pseudomonas* spp. in their research, and all *Pseudomonas* spp. were found to be 100% resistant to amoxicillin, similar to our study. Naik and Deepak (2016) also investigated *E. coli, Pseudomonas* spp., *Enterobacter* spp., and *S. aureus* from the effluent of the pharmaceutical industry in their study. They reported that most isolates were multi-drug resistant against amoxicillin, cloxacillin, cefotaxime, and penicillin. Al-Bahry et al. (2014) determined that wastewater from hospitals, pharmaceutical factories, and agricultural runoff contaminates surface and groundwater by introducing various antibiotic-resistant bacteria (MARB) and antimicrobial medicines.

Hospital wastewater (HWW) is quite different from the wastewater discharged from other sources and is hazardous and infectious. The HWW with many microbes and emerging infectious particles such as prions, viroids, and toxins is hazardous for the environment, and ultimately human health. The research on HWW has revealed that the most predominant pathogenic bacteria found are of the genus *Bacillus*, which count for 80–90% with *Staphylococcus* and *Streptococcus* varying from 5 to 10%. *E. coli* and *P. aeruginosa* are also commonly found in HWW along with other nosocomial pathogens including *Candida albicans*, *Klebsiella, Proteus*, and *Enterobacter* species. The wastewater coming from the laboratories and hospitals also consists of multiple microbes with drug resistance properties including Acinetobacter, Enterococcus, and Pseudomonas species. *S. aureus* is the most common pathogenic gram-positive bacterium with a high level of MDR (Picão et al., 2013; Kaur et al., 2020). The fourth industrial revolution, commonly known as Industry 4.0, has significantly influenced academia, government policymaking, and the industrial sector. Extensive research and analysis are being conducted to explore the potential of Industry 4.0 in enhancing various aspects such as business models, product quality, employee skills, communication, and supply chains (Sriram, 2022). “Industry 4.0” encompasses a set of crucial enabling technologies, including cyber-physical systems, the Internet of Things (IoT), artificial intelligence (AI), and big data analytics, which incorporate embedded devices. These components play a vital role in the transformation of industrial environments into mechanized and digitized settings (Uwamungu et al., 2022). So the use of AI methodologies applied to water treatment processes and desalination, optimizing these processes and offering practical solutions to challenges related to water scarcity and pollution (Uwamungu et al., 2022).

Previous research reported that their findings are similar to our present study. The present study’s result indicates that all three different types of bacteria were commonly present in pharmaceutical effluents. According to the results, *P. aeruginosa* was the most predominant isolate that carried resistant genes, transferred to environmental wastages, and finally transferred resistant genes to human beings. These antibiotic-resistant bacteria cause disease in human beings. The study concluded that Gram-positive and Gram-negative isolates of bacteria prevail in wastewater sources like our research sites and are predicted to be resistant to antimicrobial drugs associated with the industry’s contaminants. Although the isolated isolates of bacteria possess the ability to persist in that hostile environment, there are still many chances for their spreading externally via different routes, which could threaten the healthcare choices available. Before discharging waste into the environment, it is essential to prevent pollution and the spread of resistant bacteria pathogens through preprocessing.

## Conclusion

Pharmaceutical wastewater treatment should minimize antibiotic residues, heavy metals, sediments, and harmful microorganisms or convert them into beneficial ecological forms. Microorganisms that are not the primary cause of infection may spread antibiotic resistance to other microbes, especially human infections, posing a public health risk. Antibiotics and antibiotic-resistant microorganisms have been found to enter the environment. Antibiotic resistance in bacteria from different environments is rising globally. More environmental samples have demonstrated antibiotic-resistant bacteria than pre-antibiotic microbes. Stopping untreated effluent of pharmaceuticals into the environment and improving effluent treatment technologies should be appropriate techniques to remove antibiotic and multiple antibiotics resistant bacteria from pharmaceutical wastewater. Although, pharmaceutical effluents are a crucial area of research to understand and mitigate the spread of antibiotic resistance but the genotypic analysis, like whole-genome sequencing although cost-prohibitive and labor-intensive but sequencing and comparing to databases can help and can lead to a conclusive identification.

## Data availability statement

The 16S rRNA sequence data has been submitted to GenBank/DDBJ/ENA under accession no. OR029393.

## Author contributions

MM: Conceptualization, Writing – original draft. MAH: Formal analysis, Writing – original draft. MIH: Formal analysis, Writing – original draft. MS: Methodology, Writing – original draft. NAR: Validation, Supervision, Writing – original draft. MKH: Writing – original draft, Writing – review & editing. MD: Investigation, Writing – original draft. H-AN: Validation, Writing – original draft. TD: Writing – original draft, Writing – review & editing. MI: Investigation, Writing – original draft. MB: Writing – original draft, Writing – review & editing.
