# Supplementary material for: Isolation, identification, and characterization of resistant bacteria to antibiotics from pharmaceutical effluent and study of their antibiotic resistance
Source: Front Microbiol. 2023 Dec 27;14:1307291. doi: 10.3389/fmicb.2023.1307291 (PMC10790235; doi:10.3389/fmicb.2023.1307291)
Supplement: Supplementary file 1 [file Data_Sheet_1.DOCX]

**Table S1. 16s RNA sequence Pseudomonas aeruginosa strain S1C1 16S ribosomal RNA gene, partial sequence**

| GenBank: OR029393.1  >OR029393.1 Pseudomonas aeruginosa strain S1C1 16S ribosomal RNA gene, partial sequence  ACTGATAAGCATCCGGTTGAAGAGTTCCGATTAGCCCTAGTGAGAGAATCCAGTAGTAATCTTGCGTTGA  CGTACAAACAGAATAGGCACGGCTAACTCGGCAGCAGGCGCGGTAATCGAGGTGCAGCGTTATCGGATTA  CTGGGCGTAAGCGCGCGTAGGTGGTTCAGCAAGTTGGATGTGAAATCCCCGGGCTCAACCTGGGAACTGC  ATCCAAAACTACTGAGCTAGAGTACGGTAGAGGGTGGTGGAATTTCCTGTGTAGCGGTGAAATGCGTAGA  TATAGGAAGGAACACCAGTGGCGAAGGCGACCACCTGGACTGATACTGACACTGAGGTGCGAAAGCGTGG  GGAGCAAACAGGATTAGATACCCTGGTAGTCCACGCCGTAAACGATGTCGACTAGCCGTTGGGATCCTTG  AGATCTTAGTGGCGCAGCTAACGCGATAAGTCGACCGCCTGGGGAGTACGGCCGCAAGGTTAAAACTCAA  ATGAATTGACGGGGGCCCGCACAAGCGGTGGAGCATGTGGTTTAATTCGAAGCAACGCGAAGAACCTTAC  CTGGCCTTGACATGCTGAGAACTTTCCAGAGATGGATTGGTGCCTTCGGGAACTCAGACACAGGTGCTGC  ATGGCTGTCGTCAGCTCGTGTCGTGAGATGTTGGGTTAAGTCCCGTAACGAGCGCAACCCTTGTCCTTAG  TTACCAGCACCTCGGGTGGGCACTCTAAGGAGACTGCCGGTGACAAACCGGAGGAAGGTGGGGATGACGT  CAAGTCATCATGGCCCTTACGGCCAGGGCTACACACGTGCTACAATGGTCGGTACAAAGGGTTGCCAAGC  CGCGAGGTGGAGCTAATCCCATAAAACCGATCGTAGTCCGGATCGCAGTCTGCAACTCGACTGCGTGAAG  TCGGAATCGCTAGTAATCGTGAATCAGAATGTCACGGTGAATACGTTCCCGGGCCTTGTACACACCGCCC  GTCACACCATGGGAGTGGGTTGCTCCAGAAGTAGCTAGACTAACCGCAAGGGCGRCGGTACTCAGTAGAC  GTTTKAGWG |
| --- |
|  |

|  |  |  |  |  | |  |  |
| --- | --- | --- | --- | --- | --- | --- | --- |
| \| **Table S2: MAR Index of *Pseudomonas aeruginosa* (5)** \| \| \| \| \| \| --- \| --- \| --- \| --- \| --- \| \|  \|  \|  \|  \|  \| \| Isolate No. \| No. of antibiotics to which  isolates was resistant (a) \| No. of antibiotics to which  isolates was used (b) \| MAR Index =a/b \|  \| \| S1C1 \| 12 \| 12 \| 1.00 \|  \| \| S1C2 \| 12 \| 12 \| 1.00 \|  \| \| S1C3 \| 12 \| 12 \| 1.00 \|  \| \| S1C4 \| 12 \| 12 \| 1.00 \|  \| \| S1C5 \| 11 \| 12 \| 0.92 \|  \|  \| **Table S3: MAR Index of *Bacillus* spp. (4)** \| \| \| \| \| \| --- \| --- \| --- \| --- \| --- \| \|  \|  \|  \|  \|  \| \| Isolate No. \| No. of antibiotics to which  isolates was resistant (a) \| No. of antibiotics to which  isolates was used (b) \| MAR Index =a/b \|  \| \| S1C2 \| 12 \| 12 \| 1.00 \|  \| \| S2C3 \| 10 \| 12 \| 0.833 \|  \| \| S2C4 \| 11 \| 12 \| 0.92 \|  \| \| S3C1 \| 12 \| 12 \| 1.00 \|  \| \|  \|  \|  \|  \|  \|  \| **Table S4: MAR Index of *Staphylococcus* spp. (3)** \| \| \| \| \| \| --- \| --- \| --- \| --- \| --- \| \|  \|  \|  \|  \|  \| \| Isolate No. \| No. of antibiotics to which  isolates was resistant (a) \| No. of antibiotics to which  isolates was used (b) \| MAR Index =a/b \|  \| \| S2C3 \| 12 \| 12 \| 1.00 \|  \| \| S4C1 \| 12 \| 12 \| 1.00 \|  \| \| S4C2 \| 12 \| 12 \| 1.00 \|  \|   **Table S5: Calculation of Zone of Inhibition of *Pseudomonas* aeruginosa, *Bacillus* spp. and *Staphylococcus* spp.** | | | | | | | |
|  |  |  |  |  | |  |  |
| Antimicrobial agent with disc cons (μg) | *Pseudomonas* spp. | | *Bacillus* spp. | | *Staphylococcus* spp. | | |
|  | Zone of inhibition (mm) | | Zone of inhibition (mm) | | Zone of inhibition (mm) | | |
| Cloxacillin (30) | 0 | | 0 | | 0 | | |
| Amoxicillin (30) | 0 | | 0 | | 0 | | |
| Cefixime (30) | 0 | | 5 | | 0 | | |
| Vancomycin (30) | 0 | | 16 | | 0 | | |
| Ciprofloxacin (30) | 30 | | 0 | | 0 | | |
| Penicillin (10) | 0 | | 0 | | 0 | | |
| sulphamethoxazole (25) | 0 | | 0 | | 0 | | |
| Chloramphenicol (30) | 0 | | 20 | | 0 | | |
| Tobramycin (10) | 0 | | 0 | | 0 | | |
| Cefotaxime (30) | 0 | | 0 | | 0 | | |
| Caphalexin (30) | 0 | | 0 | | 0 | | |
| Cefradin (30) | 0 | | 8 | | 0 | | |
|  |  |  |  |  | |  |  |
|  |  |  |  |  | |  |  |
